# Reduced blood glucose levels by the combination of vadadustat in an elderly patient with chronic kidney disease who was receiving mitiglinide and sitagliptin: a case report

**DOI:** 10.1186/s40780-023-00316-8

**Published:** 2023-12-11

**Authors:** Ayumi Takakura, Toshinori Hirai, Naomi Hamaguchi, Rika Mukohara, Kazutaka Matsumoto, Yutaka Yano, Takuya Iwamoto

**Affiliations:** 1 Department of Pharmacy, Mie University Hospital, 2-174 Edobashi, Tsu, Mie 514-8507 Japan; 2 Department of Diabetes and Endocrinology, Mie University Hospital, 2-174 Edobashi, Tsu, Mie 514-8507 Japan

**Keywords:** Vadadustat, Sitagliptin, Mitiglinide, Organic anion transporter 3, Drug-drug interaction, Hypoglycemia

## Abstract

**Background:**

Our case is the first report showing the development of hypoglycemia following the administration of vadadustat in a patient with chronic kidney disease being treated with mitiglinide and sitagliptin, possibly due to drug–drug interaction between vadadustat and sitagliptin under the administration of mitiglinide.

**Case presentation:**

A 72-year-old man with type 2 diabetes mellitus had received sitagliptin 50 mg once daily and mitiglinide 10 mg three times daily over the last 3 years. He initiated vadadustat 300 mg once daily orally on day X owing to renal anemia (hemoglobin A1c: 7.4% and estimated glomerular filtration rate: 28.0 mL/min/1.73 m^2^). On day 23, he developed hypoglycemia with a blood glucose level of 67 mg/dL. The mean blood glucose level ± standard deviation was lower in the first 24 days of co-administration of vadadustat (before breakfast: 94 ± 14 mg/dL, before lunch: 109 ± 24 mg/dL, and before dinner: 126 ± 39 mg/dL) than in the last 2 weeks (before breakfast: 108 ± 14 mg/dL, before lunch: 122 ± 24 mg/dL, and before dinner: 158 ± 39 mg/dL). Considering the timing of the concomitant administration of vadadustat, hypoglycemia may have been caused by the drug–drug interaction between sitagliptin and vadadustat, and he discontinued treatment with vadadustat. The mean blood glucose levels improved two weeks after the discontinuation of vadadustat (before breakfast: 121 ± 25 mg/dL, before lunch: 147 ± 38 mg/dL, and before dinner: 161 ± 36 mg/dL). The drug interaction probability scale was classified as "Probable" (5 points).

**Conclusions:**

Hypoglycemia was observed when sitagliptin, mitiglinide, and vadadustat were concomitantly administered, which may have resulted in a drug–drug interaction between vadadustat and sitagliptin via OAT3 inhibition in the renal tubules.

## Background

Vadadustat, an oral hypoxia-inducible factor–prolyl hydroxylase (HIF-PHD) inhibitor, stabilizes HIF and stimulates endogenous erythropoietin by mimicking hypoxia, which increases hemoglobin levels in patients with renal anemia [1]. HIF-PHD inhibitors are not inferior in efficacy and tolerability to recombinant human erythropoietin-stimulating agents (ESA) [1]. However, several studies have warned about the pharmacokinetic drug–drug interaction between HIF-PHD inhibitors and inhibitors/inducers of drug-metabolizing enzymes and transporters (e.g., cytochrome P450 2C8 and organic anion transporter [OAT] 3) [2].

Vadadustat is a substrate of OAT1 and OAT3 localized to the renal tubular cell and acts as an inhibitor of OAT3. The Japanese package insert of VAFSEO® Tablet (Mitsubishi Tanabe Pharma Corporation, Osaka, 3rd edition, revised in September 2021) indicated that vadadustat would interact with OAT3 substrates such as furosemide and methotrexate. As sitagliptin, one of the OAT3 substrates, is often prescribed for the treatment of type 2 diabetes mellitus complicated by chronic kidney disease and renal anemia, drug interactions with vadadustat are of concern.

In this report, we describe a case of hypoglycemia in an elderly patient with chronic kidney disease and type 2 diabetes, possibly due to drug–drug interaction between vadadustat and sitagliptin under the administration of mitiglinide.

## Case presentation

A 72-year-old man (height 168.5 cm, weight 72.4 kg, and body mass index 25.5 kg/m^2^) had type 2 diabetes mellitus and stage 4 chronic kidney disease (estimated glomerular filtration rate [eGFR] 28.0 mL/min/1.73 m^2^) before X-47 years. Other medical history included heart failure with reduced ejection fraction due to acute myocardial infarction, right lower extremity atherosclerosis obliterans, cataracts, and osteoporosis. He had no family history of diabetes, and no history of allergies and side reactions.

He underwent an emergent percutaneous coronary intervention at Mie University Hospital in X-5 years but did not achieve good glycemic control despite taking glimepiride 3 mg once daily, sitagliptin 50 mg once daily, and metformin 250 mg twice daily (fasting blood glucose level 327 mg/dL and hemoglobin A1c [HbA1c] 7.8%). His primary physician changed his antidiabetic medication to sitagliptin 50 mg once daily, mitiglinide 10 mg three times daily, and insulin glargine 10 units once daily, and he was subsequently discharged from the hospital for regular visits.

**Fig. 1:**
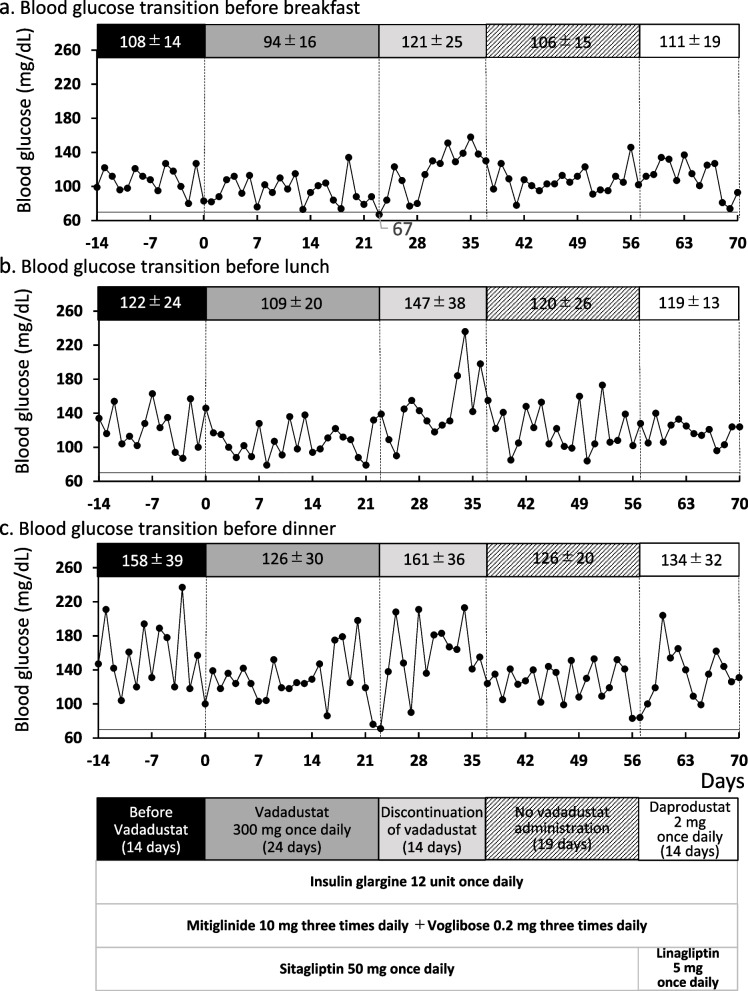
Summary of blood glucose transition and treatment course. The x-axis indicates the number of days and day 0 indicates the day of the initiation of vadadustat. The y-axis indicates fasting blood glucose before breakfast (**a**), before lunch (**b**), and before dinner (**c**). Closed circles show the self-monitoring of blood glucose. Solid lines indicate a blood glucose level of 70 mg/dL, defined as hypoglycemia. On day 23, the patient developed hypoglycemia with a blood glucose level of 67 mg/dL before breakfast. Black, gray, light gray, and white bars represent the period before vadadustat (days -14 to -1), concomitant with vadadustat (days 0 to 23), discontinuation of vadadustat (days 24 to 37), and concomitant with daprodustat (days 57 to 70). Hatched bar represents the period between vadadustat discontinuation and daprodustat initiation (days 38 to 56). Each value represents the blood glucose mean (± standard deviation) during indicated periods. Bars below the graphs represent the administration of hypoglycemic drugs (insulin glargine, mitiglinide, voglibose, sitagliptin, and linagliptin) and HIF-PHD inhibitors (vadadustat and daprodustat)

The patient’s daily dose of insulin glargine was increased from 10 to 12 units because of poor glycemic control (X-4 years; HbA1c 7.9%). Nevertheless, he did not obtain good glycemic control in X-3 years (HbA1c 8.2%). His primary physician confirmed negative findings of anti-glutamic acid decarboxylase antibody, C-peptide level of 2.9 ng/mL, and C-peptide index of 1.6. In X-2 years, voglibose 0.2 mg three times daily was added to the present regimen (HbA1c 8.1%). In X year (day 0), he orally received vadadustat 300 mg once daily with a diagnosis of renal anemia (hemoglobin 9.9 g/dL and HbA1c 7.4%). His eGFR was approximately 30 mL/min/1.73 m^2^ during the follow-up (Table 1). The blood glucose mean (± standard deviation) over the last two weeks (days -14 to -1) was 108 ± 14 mg/dL before breakfast, 122 ± 24 mg/dL before lunch, and 158 ± 39 mg/dL before dinner (Fig. 1). The prescribed medications on day 0 were sitagliptin 50 mg once daily, mitiglinide 10 mg three times daily, voglibose 0.2 mg three times daily, insulin glargine injection 12 units once daily, aspirin enteric tablets 100 mg once daily, rosuvastatin 10 mg once daily, esomeprazole 20 mg once daily, furosemide 20 mg once daily, carvedilol 10 mg twice daily, eplerenone 25 mg once daily, perindopril 2 mg once daily, and minodronic acid 50 mg every 4 weeks. There were no significant changes in medication history and lifestyle habits, such as diet and exercise, during treatment with vadadustat. Self-monitoring of blood glucose showed a decreasing tendency on day 18 after the start of vadadustat administration. He developed asymptomatic hypoglycemia on day 23 (Fig. 1, blood glucose level 67 mg/dL). The blood glucose level of the concomitant vadadustat period (days 0 to 23) was 94 ± 16 mg/dL before breakfast, 109 ± 20 mg/dL before lunch, and 126 ± 30 mg/dL before dinner (Fig. 1). He called his outpatient attending physician and visited the hospital on the same day. This phenomenon was considered to be a result of the drug–drug interaction between sitagliptin and vadadustat via OAT3 inhibition, resulting in an enhanced hypoglycemic effect of sitagliptin and mitiglinide.

**Table 1:** Clinical laboratory data during the treatment of vadadustat

	Unit	Before the initiation of vadadustat (day -49)	At the initiation of vadadustat (day 0)	At the initiation of daprodustat (day 57)
Total protein	g/dL	7.2	7.1	7.6
Albumin	g/dL	3.9	4.1	4.0
BUN	mg/dL	40.4	44.1	43.2
Creatinine	mg/dL	1.91	1.79	1.95
eGFR	mL/min/1.73m^2^	28.0	30.1	27.4
Na	meq/L	137	139	136
K	meq/L	5.5	5.2	5.8
Cl	meq/L	105	105	103
AST	U/L	29	26	38
ALT	U/L	25	23	38
LDH	U/L	214	208	241
γ-GTP	U/L	13	12	13
T-Bil	mg/dL	0.3	0.4	0.4
Glucose	mg/dL	118	130	94
CPK	U/L	383	234	262
HDL-c	mg/dL	49.2	54.4	48.9
LDL-c	mg/dL	53	56	53
HbA1c(NGSP)	%	7.4	7.4	7.5
HbF	%	1.3	1.3	1.3
White blood cell	× 10^3^/μL	6.09	7.34	7.47
Red blood cell	× 10^3^/μL	3.26	3.28	3.43
Hemoglobin	g/dL	9.8	9.9	10.4
Hematocrit	%	30.8	30.8	32.7
MCV	fL	94.5	93.9	95.3
MCH	pg	30.1	30.2	30.3
MCHC	%	31.8	32.1	31.8
Platelet	× 10^3^/μL	161	131	152

*Abbreviations*: *BUN* blood urea nitrogen, *eGFR* estimated glomerular filtration rate, *Na* sodium, *K* potassium, *Cl* chloride, *AST* aspartate aminotransferase, *ALT* alanine aminotransferase, *LDH* lactate dehydrogenase, *γ-GTP* gamma-glutamyl transpeptidase, *T-Bil* total bilirubin, *CPK* creatinine phosphokinase, *HDL-c* high-density lipoprotein-cholesterol, *LDL-c* low-density lipoprotein-cholesterol, *HbA1c (NGSP)* hemoglobin A1c (National Glycohemoglobin Standardization Program), *HbF* fetal hemoglobin, *MCV* mean corpuscular volume, *MCH* mean corpuscular hemoglobin, *MCHC* mean corpuscular hemoglobin concentration

The blood glucose recovered to 121 ± 25 mg/dL before breakfast, 147 ± 38 mg/dL before lunch, and 161 ± 36 mg/dL before dinner after discontinuation of vadadustat (days 24 to 37) (Fig. 1). On day 56, at the regular clinic visit, his medication was changed to the alternative HIF-PHD inhibitor, daprodustat 2 mg once daily, and dipeptidyl-peptidase-4 (DPP-4) inhibitor, linagliptin 5 mg once daily, which is not transported by OAT3. Thereafter, the blood glucose remained stable at 111 ± 19 mg/dL before breakfast, 119 ± 13 mg/dL before lunch, and 134 ± 32 mg/dL before dinner (days 57 to 70). On the drug interaction probability scale (DIPS), the drug–drug interaction between sitagliptin and vadadustat was scored at 5 points, classified as “probable” [3] (Table 2).

**Table 2:** Drug interaction probability scale

Question	Patient 1
1 Are there previous credible reports of this interaction in humans?	NA (0)
2 Is the observed interaction consistent with the known interactive properties of precipitant drug?	Y (+ 1)
3 Is the observed interaction consistent with the known interactive properties of object drug?	Y (+ 1)
4 Is the event consistent with the known or reasonable time course of the interaction (onset and/or offset)?	Y (+ 1)
5 Did the interaction remit upon dechallenge of the precipitant drug with no change in the object drug?	Y (+ 1)
6 Did the interaction reappear when the precipitant drug was readministered in the presence of continued use of object drug?	NA (0)
7 Are there reasonable alternative causes for the event?	N (0)
8 Was the object drug detected in the blood or other fluids in concentrations consistent with the proposed interaction?	NA (0)
9 Was the drug interaction confirmed by any objective evidence consistent with the effects on the object drug?	Y (+ 1)
10 Was the Interaction greater when the precipitant drug dose was increased or less when the precipitant drug dose was decreased?	NA (0)
**Total scores (Causality of drug-drug interaction)**	**5 (Probable)**

*Abbreviations*: *Y* Yes, *N* No, *NA* not applicable

The aforementioned scoring procedure was proposed by Horn et al. [3]

The object drug is sitagliptin, and the precipitant drug is vadadustat

The answers and scores for each question were described. Drug interaction probability score categories are classified into four groups: doubtful (0–1), possible (2–4), probable (5–8), and highly probable (9–11)

## Discussion and conclusions

Although vadadustat is an inhibitor of OAT3 and BCRP, the drug-drug interaction with sitagliptin and vadadustat results from vadadustat-medicated OAT3 inhibition because sitagliptin is eliminated by glomerular filtration and secretion of OAT3 at the proximal tubule [4]. Vadadustat has been reported to inhibit OAT3 with a 50% inhibitory concentration (IC_50_) of 0.336 μg/mL [5]. Assuming the pharmacokinetic profile of vadadustat (free fraction; 0.2%–0.6% and the maximum blood concentration of 44.3 μg/mL at an oral dose of 300 mg) [5], the ratio of free maximum blood concentration to IC_50_ is estimated to be approximately 0.53. The International Transporter Consortium states that a free maximum blood concentration/IC_50_ ratio > 0.1 is clinically relevant for a potential drug–drug interaction [6], which supports our hypothesis that vadadustat inhibited sitagliptin renal tubular secretion via OAT3 at clinical doses. Indeed, the free maximum blood concentration/IC_50_ ratio was 1.19 based on the evidence that the free maximum blood concentration of vadadustat at an oral single dose of 450 mg is 0.4 μg/mL [7]. In contrast, the IC_50_ value of vadadustat-O-glucuronide conjugate (a main metabolite of vadadustat) at OAT3 is 9.10 μg/mL [5]. Because the free maximum blood concentration/IC_50_ ratio of vadadustat-O-glucuronate conjugate (free fraction; 12.2%–13.5% and the maximum blood concentration of 4.14 μg/mL [5]) is approximately 0.06, the interaction of vadadustat-O-glucuronide conjugate with sitagliptin is unlikely. Therefore, the OAT3 mediated-drug–drug interaction with sitagliptin was probably due to vadadustat and not to vadadustat-O-glucuronate conjugate. From a pharmacological viewpoint, previous reports have suggested that erythropoietin promotes the uptake of blood glucose into erythrocytes thereby decreasing blood glucose levels [8, 9]. Therefore, co-administration of HIF-PHD inhibitor may increase erythropoietin level and alter blood glucose levels.

A total clearance of sitagliptin is around 400 mL/min, where approximately 80% of sitagliptin is excreted by glomerular filtration and tubular secretion via OAT3 [4]. Therefore, vadadustat can inhibit OAT3-mediated renal tubular secretion and accumulate sitagliptin in the body. Sitagliptin dose-dependently inhibits DPP-4, thereby elevating GLP-1 and GIP, which promote insulin secretion on pancreatic beta cells, leading to weak hypoglycemic action. Many studies have shown that DPP-4 inhibitors complementarily increase hypoglycemic risk when administered with sulfonylureas, which strongly secrete insulin independent of blood glucose levels [10]. In particular, chronic kidney disease is a common risk factor for hypoglycemia in the combination uses of DPP-4 inhibitors and sulfonylurea drugs [11]. In general, glinides and sulfonylureas have the same pharmacologic action of insulinotropic effect. However, glinides exhibit faster hypoglycemic action and a lower risk of hypoglycemia because of their rapid binding and dissolution to sulfonylurea receptors [12]. Nevertheless, the risk of hypoglycemia is higher with the combination of sitagliptin and glinide than with glinide alone (4.0% vs. 1.3%) [13]. Mitiglinide undergoes hepatic metabolism via uridine diphosphate glucuronosyltransferase (UGT)1A9 and UGT1A3 [14]. Additionally, the apparent clearance of mitiglinide is reduced in chronic kidney disease, resulting in increased exposure to mitiglinide [14]. Similarly, sitagliptin has been reported to increase the area under the blood concentration–time curve in patients with chronic kidney disease [15]. Therefore, chronic kidney disease contributes to high exposure to sitagliptin and mitiglinide and increases the risk of hypoglycemia.

There is a literature demonstrating that tubular secretory clearance is expected to be reduced, especially in severe chronic kidney disease [16]. Accordingly, co-administration of vadadustat should inhibit the residual activity of OAT3 and lower the tubular secretion clearance of sitagliptin, which may enhance the pharmacological effect of vadadustat.

In summary, hypoglycemia was observed when sitagliptin, mitiglinide, and vadadustat were concomitantly administered, which may have resulted in a drug–drug interaction between vadadustat and sitagliptin via OAT3 inhibition. We consider that the main cause of the hypoglycemia in this case is the combination of sitagliptin and mitiglinide. This potential drug–drug interaction requires special attention in patients with impaired renal function, and the selection of a HIF-PHD inhibitor based on pharmacokinetic characteristics to prevent drug–drug interactions.

## Data Availability

Data sharing is not applicable to this article as no datasets were generated or analysed during the current study.

## Abbreviations

HIF-PHD: Hypoxia-inducible factor–prolyl hydroxylase
ESA: Erythropoietin-stimulating agents
OAT: Organic anion transporter
eGFR: Estimated glomerular filtration rate
HbA1c: Hemoglobin A1c
DPP-4: Dipeptidyl-peptidase-4
DIPS: Drug interaction probability scale
UGT: Uridine diphosphate glucuronosyltransferase
IC_50_: 50% Inhibitory concentration
